# Clinical Risk Characteristics of Upper Gastrointestinal Hemorrhage Severity: A Multivariable Risk Analysis

**DOI:** 10.4021/gr463w

**Published:** 2012-07-20

**Authors:** Rangson Chaikitamnuaychok, Jayanton Patumanond

**Affiliations:** a Department of Surgery, Kamphaeng Phet Hospital, Kamphaeng Phet, 62000, Thailand; b Clinical Epidemiology Unit, Faculty of Medicine, Chiang Mai University, Chiang Mai, 50200, Thailand

**Keywords:** Upper gastrointestinal hemorrhage, Severity, Stratification, Clinical risk, Prognostic indicators, Multivariable analysis

## Abstract

**Background:**

Upper gastrointestinal hemorrhage (UGIH) is one of the common clinical manifestations encountered in most emergency departments. Patient characteristics indicating UGIH severity in developing countries may be different from those in developed countries. The present study was designed to explore clinical prognostic indicators for UGIH severity.

**Methods:**

A retrospective cohort study was conducted in a university affiliated tertiary hospital in Kamphaeng Phet, Thailand. Medical folders of patients with UGIH were reviewed. Patients were grouped into 3 severity levels, based on criteria proposed by The American College of Surgeon. Pre-defined prognostic indicators were compared. The prognostic indicators for UGIH severity were analyzed by a multivariable continuation ratio ordinal logistic regression and presented with odds ratios.

**Results:**

From 1,043 eligible medical folders, 984 (94.3%) complete folders were used in analysis. There were 241, 631 and 112 patients in the mild, moderate and severe UGIH groups. Six independent indicators of severe UGIH were, hemoglobin < 100 g/dL (OR = 13.82, 95% CI = 9.40 to 20.33, P < 0.001), systolic blood pressure < 100 mmHg (OR = 11.01, 95% CI = 7.41 to 16.36, P < 0.001), presence of hepatic failure (OR = 5.50, 95% CI = 1.14 to26.64, P = 0.037), presence of cirrhosis (OR = 2.03, 95% CI = 1.32 to 3.11, P = 0.001), blood urea nitrogen ≥ 35 mmol/L (OR = 1.73, 95% CI = 1.25 to 2.40, P = 0.001), and pulse rate ≥ 100 per minute (OR = 1.72, 95% CI = 1.21 to 2.45, P = 0.003).

**Conclusions:**

Pulse rate ≥ 100 per minute, systolic blood pressure < 100 mmHg, hemoglobin < 10 g/dL, blood urea nitrogen ≥ 35 mmol/L, presence of cirrhosis and presence of hepatic failure are prognostic indicators for an increase in UGIH severity levels. They are potentially useful in UGIH risk stratification.

## Introduction

Upper gastrointestinal hemorrhage (UGIH) is one among the common clinical manifestations encountered in most emergency departments. The annual incidence varies from 50 to 150 episodes per 100,000 populations [1], with 11-14% case fatality [2, 3]. Mortalities from UGIH increase in elderly patients and in patients with co-morbidities [4, 5]. Advances in medical technology, particularly endoscopic instrumentations, in the past 10 years do not seem to decrease case fatality from UGIH [2, 3, 5]. The costs of patient care are still very high in the United States, approximately 750 million US dollars per year [6].

Usual managements of patients with UGIH began with patients screening, clinical assessment and evaluation, resuscitation, endoscopy [7] and surgical intervention when indicated.

Endoscopy is essential and is the key measures to evaluate the risks of re-bleeding and mortality. At the same time, endoscopy is valuable for the pathological and anatomical diagnosis, patient risk stratification and hemostasis [8]. Endoscopy is usually recommended within 24 hours after the patient vital signs are stabilized. Although endoscopy is very accurate in patient risk stratification [9], it may not be performed within the first 24 hours in all patients with UGIH, especially in areas with limitations in health resources and/or medical personnel. Studies over the past 10 years were focused on how to triage UGIH cases into categories such as emergent, semi-elective or as out-patients, in order to define appropriate time for endoscopy, but the results are still in-conclusive [8].

Various clinical risk characteristics were adopted in the process of patient assessment. These characteristics included the patient age, presence of shock, professional diagnosis, hemoglobin level measured on arrival at emergency rooms, presenting symptoms, size of ulcers, stigmata of hemorrhage and blood transfusion [10-20]. Those clinical risks were further applied into a prognostic scoring algorithm to evaluate UGIH patients risk of death and/or re-bleeding [7], and also to screen for and to select high risk patients into intervention within an appropriate time.

The most well known prognostic scores are probably “The Rockall Score” [21] and “The Blatchford Score” [22]. The Rockall Score was developed in the UK in 1995 [2], published in 1996 [3] and was validated in the following year [23]. The objective of the score was to predict patient poor clinical outcomes. It was also validated in many other settings, but with diverse conclusions. Some authors reported good prediction for re-bleeding, but poor prediction for death [24, 25]. The others reported the opposite directions [26, 27]. However, all authors agreed that a decrease in scores results in an over-estimation and that an increase in scores results in a loss of discrimination [7]. The Blatchford Score was proposed in 2000 [22] to be used in patients evaluation before endoscopy, and to search for patients requiring intervention, such as blood transfusion, hemostasis, either with endoscopy and/or surgery [22]. The score comprised both clinical examinations and laboratory tests [2, 22]. Validation studies of the score concluded that it can be used in screening UGIH patients on admission into those with high risk who needed blood transfusion, endoscopy or intervention, and patients with low risk who do not need such interventions [28-30]. External validation of the Blatchford score reported a high sensitivity of 99-100% in high risk patients [29, 30], but a low specificity of 13% [30]. Other prognostic scores such as “The Baylor College Score” [31] and “The Cedars-Sinai Score” [32] were not worldwide, partly because they required early endoscopy and that re-bleeding was unacceptably under-estimated [7].

The clinical risk characteristics reported in developed countries may be different from developing countries. The present study was initiated to explore and investigate for the different clinical risk characteristics associated with the severity of UGIH in a tertiary hospital in a developing country

## Methods

### Patients

Kamphaeng Phet Hospital is a university affiliated tertiary hospital located in the lower northern part of Thailand. It represents a common tertiary hospital of the country. Medical folders of patients admitted to the hospital with UGIH during 2009 - 2010 were searched from the hospital computer database system, using ICD-10, K-920-hematemesis, K-921-melena, and K-922-gastro intestinal hemorrhage unspecified. The number of eligible patients under the above criteria was 685 in 2009 and 350 in 2010.

### Risk characteristics

Study parameters included: 1) Demographic data; gender and age; 2) Mode of presentation; hematemesis, coffee ground vomiting, hematochezia, melena and syncope; 3) Hemodynamic; pulse rate and systolic blood pressure; 4) Biochemical investigations; hemoglobin and blood urea nitrogen; 5) Co-morbidities; presence of cirrhosis, hepatic failure, cardiac failure and renal failure.

### Definitions of severity

The severity of UGIH was operationally defined by the following criteria: 1) Severe; patients who hemostasis was done under surgical intervention, patients with grade 3 and 4 shock (Table 1) [33], or patients who died of UGIH; 2) Moderate; patients who hemostasis was done under endotherapy (endoscopy), patients with re-bleeding, patients with grade 1 and 2 shock [33], or patients who required blood transfusion; 3) Mild; patient without shock, patients who did not require any intervention under endoscopy, or patients who required no blood transfusion.

**Table 1:** Classification of Hypovolemic Shock Manifestation and Fluid Resuscitation

	Class I	Class II	Class III	Class IV
Blood loss (mL)	Up to 750	750 - 1,500	1,500 - 2,000	> 2,000
Blood loss (%BV*)	Up to 15%	15-30%	30-40%	> 40%
Pulse rate (/min)	< 100	100 - 120	120 - 140	> 140
Blood pressure (mmHg)	Normal	Normal	Decreased	Decreased
Pulse pressure (mmHg)	Normal or increased	Decreased	Decreased	Decreased
Respiratory rate (/min)	14 - 20	20 - 30	30 - 40	> 35
Urine output (mL/h)	> 30	20 - 30	5 - 15	Negligible
CNS**/mental status	Slightly anxious	Mildly anxious	Anxious and confused	Confused and lethargic
Fluid replacement	Crystalloid	Crystalloid	Crystalloid and blood	Crystalloid and blood

*BV: blood volume; **CNS: central nervous system. Abstracted and simplified from The American College of Surgeons, 2008 [33].

### Data analysis

Patient parameters were compared across the 3 severity categories by chi-squared tests for trend and a two-way analysis of variance by rank (Friedmann ANOVA). To maximize the statistical power of analysis of the 3 ordinal clinical outcomes (severe, moderate and mild), we chose a multivariable ordinal continuation ratio logistic regression to analyze the prognostic indicators for UGIH severity.

The study protocol was approved by The Kamphaeng Phet Hospital Ethical Committee for Clinical Research. Patient consent forms were not required in this retrospective data review. Patient names and other traceable identifications were confidential and omitted in all process of data management.

The 2 authors received no outside grants and reported no conflicts of interests.

## Results

During 2009 - 2010, there were 1,043 patients who met the study criteria. Some information was missing in 59 patients. These missing information were; pulse rate (n = 8), systolic blood pressure (n = 6), hemoglobin (n = 20) and blood urea nitrogen (n = 47). The remaining patients (n = 984, 94.3%) were used in analysis.

Patients were categorized into 3 levels of severity; mild (n = 241), moderate (n = 631) and severe (n = 112) following the aforementioned criteria. The three severity groups were similar in gender, presentation with hematemesis, and hematochezia (Table 2). The characteristics that were different among the three groups were; age, presentation with coffee ground vomiting, melena, syncope, pulse rate, systolic blood pressure, hemoglobin level, blood urea nitrogen, presence of cirrhosis, hepatic failure, cardiac failure, and renal failure.

**Table 2:** Demographic and Clinical Profiles of Patients With Upper Gastrointestinal Hemorrhage, by Severity Definition

Characteristics	Mild (n = 241)	Moderate (n = 631)	Severe (n = 112)	P-value*
	n (%)	n (%)	n (%)	
Demographics				
Male	164 (68.1)	408 (64.7)	79 (70.5)	0.968
Age (year) (mean ± SD)	54.4 ± 17.9	60.3 ± 14.7	58.1 ± 13.8	0.009
Mode of presentation				
Hematemesis	111 (46.1)	286 (45.3)	58 (51.8)	0.467
Coffee ground vomiting	63 (26.1)	110 (17.4)	23 (20.5)	0.051
Hematochezia	19 (7.9)	38 (6.0)	8 (7.1)	0.586
Melena	108 (44.8)	401 (63.6)	65 (58.0)	< 0.001
Syncope	26 (10.8)	136 (21.6)	30 (26.8)	< 0.001
Hemodynamics				
Pulse (/min) (mean ± SD)	89.8 ± 16.6	91.6 ± 15.6	93.4 ± 16.5	0.024
SBP (mmHg) (mean ± SD)	128.0 ± 20.9	120.8 ± 20.6	89.4 ± 16.5	< 0.001
Biochemicals				
Hemoglobin (g/dL) (mean ± SD)	11.4 ± 2.4	7.0 ± 2.1	7.4 ± 2.8	< 0.001
BUN (mmol/L) (mean ± SD)	23.7 ± 16.2	36.6 ± 21.7	37.5 ± 22.6	< 0.001
Co-morbidities				
Cirrhosis	14 (5.8)	101 (16.0)	26 (23.2)	< 0.001
Hepatic failure	0 (0)	6 (1.0)	2 (1.8)	0.064
Cardiac failure	0 (0)	6 (1.0)	3 (2.7)	0.017
Renal failure	4 (1.7)	53 (8.4)	12 (10.7)	< 0.001
Clinical outcomes				
Rebleed	6 (2.5)	41 (6.5)	17 (15.2)	< 0.001
Dead	0 (0)	1 (0.2)	19 (17.0)	< 0.001

*P-value from chi-squared for trend (categorical data), or two-way ANOVA by rank (numerical data).

The significantly different characteristics detected in Table 2 were further analyzed simultaneously to explore for their independent effects, by a multivariable ordinal continuation ratio logistic regression. The remaining independent prognostic indicators for UGIH severity were; age ≥ 60 years, presentation with melena, syncope, pulse rate ≥ 100 per minute, systolic blood pressure < 100 mmHg, hemoglobin < 10 g/dL, blood urea nitrogen ≥ 35 mmol/L, presence of cirrhosis, hepatic failure, cardiac failure, and renal failure. The strongest parameters were; hemoglobin < 10 g/dL (odds ratio = 13.82, 95% confidence interval; 9.40 to 20.33, P < 0.001) and systolic blood pressure < 100 mmHg (odds ratio = 11.01, 95% confidence interval; 7.41 to 16.36, P < 0.001) (Table 3).

**Table 3:** Risk of Increasing in Severity of Upper Gastrointestinal Hemorrhage (Odds Ratio and 95% Confidence Interval), by Demographic and Clinical Profiles

Characteristics	OR*	95%CI	P-value
Demographics			
Male	0.99	0.73 - 1.36	0.996
Age (year)			
< 60	1.00	ref	
≥ 60	1.27	0.94 - 1.73	0.120
Mode of presentation			
Hematemesis	1.30	0.93 - 1.84	0.129
Coffee ground	0.87	0.57 - 1.31	0.500
Hematochezia	0.87	0.47 - 1.59	0.651
Melena	1.10	0.79 - 1.53	0.574
Syncope	1.29	0.89 - 1.87	0.179
Hemodynamics			
Pulse (/min)			
< 100	1.00	ref	
≥ 100	1.72	1.21 - 2.45	0.003
SBP (mmHg)			
< 100	11.01	7.41 - 16.36	< 0.001
≥ 100	1.00		
Biochemicals			
Hemoglobin (g/dL)			
< 10	13.82	9.40-20.33	< 0.001
≥ 10	1.00	ref	
BUN (mmol/L)			
< 35	1.00	ref	
≥ 35	1.73	1.25 - 2.40	0.001
Co-morbidities			
Cirrhosis	2.03	1.32-3.11	0.001
Hepatic failure	5.50	1.14 - 26.64	0.034
Cardiac failure	4.07	0.83 - 19.90	0.083
Renal failure	1.26	0.68 - 2.35	0.466

*Odds ratio from multivariable ordinal continuation ratio logistic regression.

## Discussion

The prognostic indicators for UGIH severity detected in our study agreed with findings in the previous studies of prognostic score [13, 19], such as The Rockall Score (age, systolic blood pressure, pulse rate, presence of hepatic failure, cardiac failure, and renal failure) [2, 3, 23], and The Blatchford Score (blood urea nitrogen, hemoglobin systolic blood pressure, pulse rate, presentation with melena, syncope, presence of liver failure, and cardiac failure) [22, 34, 35]. These indicators are straightforward and need no further explanations. However, the age parameter was different from the Blatchford’s and the other studies, proposing that the patient age [22, 34, 35] was not associated with a decision for intervention.

These similarities and discrepancies show that in developing countries, some clinical indicators detected in developed countries are similarly related to the severity of UGIH. It is worth noticing that presence of cirrhosis, the parameter not mentioned earlier in other studies, also increased the UGIH severity by 2.03 folds in our study. This may indicate that, in Thai patients, as well as in other developing countries, cirrhosis may also play an additional role in the severity of UGIH.

Patient medical files with incomplete information (5.7%) were excluded from analysis and may leave some doubts on representativeness of the study. However, we believe that the missing data were likely to be at random and that this small percentage of missing was unlikely to bias the overall findings.

The clinically and statistically important indicators detected from this study may be used in future investigations, such as to develop a prognostic scoring algorithm to screen for severity levels of patients presenting with UGIH.

### Conclusions

The pulse rate ≥ 100 per minute, systolic blood pressure < 100 mmHg, hemoglobin < 10 g/dL, blood urea nitrogen ≥ 35 mmol/L, presence of cirrhosis and hepatic failure are the risk characteristics that increase UGIH severity levels. They may guide clinicians to pay some attentions to patients with these risks. For future implications, these risk characteristics may be used in the process of UGIH risk stratification in developing countries.

## References

[1] (2002). Non-variceal upper gastrointestinal haemorrhage: guidelines.

[2] Rockall TA, Logan RF, Devlin HB, Northfield TC (1995). Incidence of and mortality from acute upper gastrointestinal haemorrhage in the United Kingdom. Steering Committee and members of the National Audit of Acute Upper Gastrointestinal Haemorrhage. BMJ.

[3] Rockall TA, Logan RF, Devlin HB, Northfield TC (1996). Risk assessment after acute upper gastrointestinal haemorrhage. Gut.

[4] Zimmerman J, Siguencia J, Tsvang E, Beeri R, Arnon R (1995). Predictors of mortality in patients admitted to hospital for acute upper gastrointestinal hemorrhage. Scand J Gastroenterol.

[5] Vreeburg EM, Snel P, de Bruijne JW, Bartelsman JF, Rauws EA, Tytgat GN (1997). Acute upper gastrointestinal bleeding in the Amsterdam area: incidence, diagnosis, and clinical outcome. Am J Gastroenterol.

[6] Jiranek GC, Kozarek RA (1996). A cost-effective approach to the patient with peptic ulcer bleeding. Surg Clin North Am.

[7] Atkinson RJ, Hurlstone DP (2008). Usefulness of prognostic indices in upper gastrointestinal bleeding. Best Pract Res Clin Gastroenterol.

[8] Hamoui N, Docherty SD, Crookes PF (2003). Gastrointestinal hemorrhage: is the surgeon obsolete?. Emerg Med Clin North Am.

[9] Rockall TA, Logan RF, Devlin HB, Northfield TC (1996). Selection of patients for early discharge or outpatient care after acute upper gastrointestinal haemorrhage. National Audit of Acute Upper Gastrointestinal Haemorrhage. Lancet.

[10] Swain CP, Kirkham JS, Salmon PR, Bown SG, Northfield TC (1986). Controlled trial of Nd-YAG laser photocoagulation in bleeding peptic ulcers. Lancet.

[11] Storey DW, Bown SG, Swain CP, Salmon PR, Kirkham JS, Northfield TC (1981). Endoscopic prediction of recurrent bleeding in peptic ulcers. N Engl J Med.

[12] Katschinski BD, Logan RF, Davies J, Langman MJ (1989). Audit of mortality in upper gastrointestinal bleeding. Postgrad Med J.

[13] Katschinski B, Logan R, Davies J, Faulkner G, Pearson J, Langman M (1994). Prognostic factors in upper gastrointestinal bleeding. Dig Dis Sci.

[14] Kalabakas A, Xourgias B, Karamanolis D (1990). Incidence and significance of stigmata of recent haemorrhage in ulcer patients without clinical evidence of recent bleeding. Gut.

[15] Wara P, Berg V, Amdrup E (1983). Factors influencing mortality in patients with bleeding ulcer. Review of 7 years' experience preceding therapeutic endoscopy. Acta Chir Scand.

[16] MacLeod IA, Mills PR (1982). Factors identifying the probability of further haemorrhage after acute upper gastrointestinal haemorrhage. Br J Surg.

[17] Bornman PC, Theodorou NA, Shuttleworth RD, Essel HP, Marks IN (1985). Importance of hypovolaemic shock and endoscopic signs in predicting recurrent haemorrhage from peptic ulceration: a prospective evaluation. Br Med J (Clin Res Ed).

[18] Branicki FJ, Coleman SY, Fok PJ, Pritchett CJ, Fan ST, Lai EC, Mok FP (1990). Bleeding peptic ulcer: a prospective evaluation of risk factors for rebleeding and mortality. World J Surg.

[19] Clason AE, Macleod DA, Elton RA (1986). Clinical factors in the prediction of further haemorrhage or mortality in acute upper gastrointestinal haemorrhage. Br J Surg.

[20] Morgan AG, Clamp SE (1984). O.M.G.E. International Upper Gastro-intestinal Bleeding Survey 1978-1982. Scand J Gastroenterol Suppl.

[21] Tham TC, James C, Kelly M (2006). Predicting outcome of acute non-variceal upper gastrointestinal haemorrhage without endoscopy using the clinical Rockall Score. Postgrad Med J.

[22] Blatchford O, Murray WR, Blatchford M (2000). A risk score to predict need for treatment for upper-gastrointestinal haemorrhage. Lancet.

[23] Rockall TA, Logan RF, Devlin HB, Northfield TC (1997). Influencing the practice and outcome in acute upper gastrointestinal haemorrhage. Steering Committee of the National Audit of Acute Upper Gastrointestinal Haemorrhage. Gut.

[24] Sanders DS, Carter MJ, Goodchap RJ, Cross SS, Gleeson DC, Lobo AJ (2002). Prospective validation of the Rockall risk scoring system for upper GI hemorrhage in subgroups of patients with varices and peptic ulcers. Am J Gastroenterol.

[25] Enns RA, Gagnon YM, Barkun AN, Armstrong D, Gregor JC, Fedorak RN (2006). Validation of the Rockall scoring system for outcomes from non-variceal upper gastrointestinal bleeding in a Canadian setting. World J Gastroenterol.

[26] Vreeburg EM, Terwee CB, Snel P, Rauws EA, Bartelsman JF, Meulen JH, Tytgat GN (1999). Validation of the Rockall risk scoring system in upper gastrointestinal bleeding. Gut.

[27] Sarwar S, Dilshad A, Khan AA, Alam A, Butt AK, Tariq S, Ahmad I (2007). Predictive value of Rockall score for rebleeding and mortality in patients with variceal bleeding. J Coll Physicians Surg Pak.

[28] Gralnek IM, Dulai GS (2004). Incremental value of upper endoscopy for triage of patients with acute non-variceal upper-GI hemorrhage. Gastrointest Endosc.

[29] Chen IC, Hung MS, Chiu TF, Chen JC, Hsiao CT (2007). Risk scoring systems to predict need for clinical intervention for patients with nonvariceal upper gastrointestinal tract bleeding. Am J Emerg Med.

[30] Masaoka T, Suzuki H, Hori S, Aikawa N, Hibi T (2007). Blatchford scoring system is a useful scoring system for detecting patients with upper gastrointestinal bleeding who do not need endoscopic intervention. J Gastroenterol Hepatol.

[31] Saeed ZA, Ramirez FC, Hepps KS, Cole RA, Graham DY (1995). Prospective validation of the Baylor bleeding score for predicting the likelihood of rebleeding after endoscopic hemostasis of peptic ulcers. Gastrointest Endosc.

[32] Hay JA, Lyubashevsky E, Elashoff J, Maldonado L, Weingarten SR, Ellrodt AG (1996). Upper gastrointestinal hemorrhage clinical—guideline determining the optimal hospital length of stay. Am J Med.

[33] Committee on Trauma, American College of Surgeons (2008). ATLS: Advanced trauma life support program for doctors.

[34] Pang SH, Ching JY, Lau JY, Sung JJ, Graham DY, Chan FK (2010). Comparing the Blatchford and pre-endoscopic Rockall score in predicting the need for endoscopic therapy in patients with upper GI hemorrhage. Gastrointest Endosc.

[35] Blatchford O, Davidson LA, Murray WR, Blatchford M, Pell J (1997). Acute upper gastrointestinal haemorrhage in west of Scotland: case ascertainment study. BMJ.

